# Physical Performance and Morphological Characteristics of Young Basketball Players before and after COVID-19

**DOI:** 10.3390/children10030493

**Published:** 2023-03-02

**Authors:** Vladan Pelemiš, Dajana Zoretić, Ivan Prskalo

**Affiliations:** 1 Faculty of Teacher Education, University of Belgrade, 11000 Belgrade, Serbia; 2 Faculty of Kinesiology, University of Zagreb, 10000 Zagreb, Croatia; 3 Faculty of Teacher Education, University of Zagreb, 10000 Zagreb, Croatia

**Keywords:** COVID-19, basketball, body composition, motor skills, adolescents

## Abstract

The aim of this study was to determine possible changes in physical performance and morphological characteristics in young basketball players before and after quarantine caused by COVID-19. The research was conducted on a total sample of 46 young basketball players. Participants were measured before the quarantine and pandemic in January 2020 and then after the quarantine period, one year later in March 2021. The results indicate the existence of significant differences in total muscle mass (*p* < 0.01) in favor of higher values measured before quarantine. Moreover, total fat percentage was higher (*p* < 0.03) after quarantine. As for physical performance, significant differences were also observed in the counter movement jump test (*p* < 0.05) and the 20 m shuttle run test (*p* < 0.05), with significantly poorer results for the group of participants measured after quarantine. The authors conclude that the consequences of the quarantine and pandemic of the SARS-CoV-2 virus were definitely negative, as higher values of body fat and lower values of the percentage of total muscle mass were observed. In addition, a lower level of the explosive power of lower limbs was observed, accompanied by lower aerobic endurance in young basketball players.

## 1. Introduction

During the pandemic of the SARS-CoV-2 virus (severe acute respiratory syndrome coronavirus (COVID-19)), WHO (World Health Organization) recommendations (2020) [1] and government measures in most countries around the world included the introduction of quarantine and restrictions on movement among people. This led to certain consequences on human anthropological status, especially for athletes, because they were deprived of their regular training process and competitions. Many countries stopped working, forcing people to stay at home and leave only in case of emergency. Research by Hammami et al. [2] has shown that this type of decision has led to negative changes in the mental composition of people. Although these restrictions have helped to reduce the rate of infection caused by this new virus, they have been found to result in negative effects on eating habits during the pandemic [3], participation in normal daily activities, programmed physical activity, and access to many other forms of recreational physical exercise [4,5]. It was also found that the measures prolonged passive use of free time with increased playing of computer games, excessive use of mobile devices, and watching television [6].

From the aspect of sports science, basketball can be defined as a complex kinesiological activity consisting of polystructural movements and a network within the internal-cooperative and external-agonistic activity, which is there to regulate the maximum efficiency of these systems [7]. Basketball is played around the world and is very popular in Europe [8], requireing good explosive power, coordination, speed and agility [9,10], specific motor/functional skills and morphological characteristics including athletic physique and body composition supported by a low percentage of fat and a higher percentage of muscle mass [11,12]. The influence of maturation on morphological characteristics and physical performance is great at this age. Young basketball players have signs of intensification and faster dynamics of growth and development of the longitudinal dimensions of the skeleton compared to the previous period. Growth in height is much more dominant than growth in width [10]. The growth of young basketball players is most intense in the period from 13 to 15–16 years, and then they grow over 10 cm. They gain approximately 7 to 10 kg of body weight, have the highest percentage of muscle mass compared to boys who do not play basketball, and up to 5%, a lower percentage fat component from 5% to 8%, higher aerobic capacity up to 20% and higher relative muscle power up to 25%. The importance of body height and body proportion in the younger categories is the basis for choosing talent in basketball. Of particular interest is the hypothetical morphological factor of longitudinal dimensionality of the skeleton, where the most important anthropometric measurements are: body height, sitting height, arm span, and leg length [13]. Moreover, motor performance can be a good indicator of success in young basketball players [14]. Some earlier studies have even shown that high demands in basketball produce major physiological and neuromuscular loads [15]. Therefore, in working with younger teams, it would be good to quantify such loads and determine the number of high-intensity activities [16], and thus ask basketball players to perform explosive activities for the lower limbs, such as sprints, jumps, accelerations, and decelerations. The sensitive period of development of explosive strength and endurance in strength begins around 8 years of life, a critical phase in the age of 7 to 17 years, where a period should be singled out, especially from 14 to 15 years. [17]. Thus, it can be concluded from the above that the selection and sports performance depends on the combination of anthropological characteristics specific to sports [18].

In the training process, for the purposes of better planning and organization of training procedures, it is important to conduct a detailed analysis of a sports discipline. Therefore, adequate motor and functional tests are very important for basketball, such as explosive power tests, which can be assessed by the counter movement jump (CMJ) test [19]. In addition, the 20 m shuttle run test [20] is very important and popular, with several names through which it is known, such as multistage fitness test, beep test, or bleep test. The test is mostly for the purpose of finding out the results of aerobic endurance [21]. When working with younger players, it is important to state that these children are at the beginning of their development period and that other factors that affect success in sport must be developed [22]. We should not forget the fact that during preadolescence, body composition (total fat and total muscle mass), in combination with growth and development factors, which are extremely susceptible to change during this period, can negatively affect physical performance and technical skills in basketball [23]. If the time of absence from the training process is added to this fact, it is considered that it can lead to a decrease in the physical and physiological parameters of basketball players [24].

One of the structural problems that sports experts have not encountered so far lies in the system of functioning of the training process of young basketball teams as a dilemma, whether the interruption, shortening, or total suspension of the training process for a long time period should (which is the case in this research) be treated as a handicap in terms of the decrease in certain anthropological characteristics? Therefore, this study should provide answers to the question of what happens to the anthropological characteristics and skills of younger players after quarantine, which caused the interruption of the normal training process for 3 months. The obtained findings could influence the planning and implementation of new training content that would be prepared in the event of a re-emergence of a similar lifestyle of athletes.

The aim of this study was to determine possible changes in morphological characteristics, motor skills, and functional ability of pioneer basketball players before and after quarantine caused by the COVID-19 virus. Based on the goal of the research, it was hypothesized that quarantine during the COVID-19 pandemic would cause qualitative changes in physical performance and morphological characteristics in basketball players aged 14 years.

## 2. Materials and Methods

The research was of a transversal nature. A quasi-experimental design was used, namely a design with non-equivalent groups and a pretest–posttest. Two measurements were performed at two time points on the same group of respondents. Parents/guardians of the participants gave their consent for their children’s participation in the research, as well as for the use of data only for scientific purposes [25].

### 2.1. Instruments

Anthropometric measurements: body height (0.1 cm)—measured with a Martin’s anthropometer according to the International biological program (IBP), body weight (0.1 kg)—was measured using InBody 230 (Biospace Co., Ltd., Seoul, Republic of Korea). Based on the measured dimensions of body weight and body height under IV nutritional status was calculated by dividing the body weight value by the squared body height: body mass index BMI (kg/m^2^)—calculated according to the Centers for Disease Control and Prevention, (2000).

Body composition was measured using four measurements: total muscle mass (0.1 kg); total amount of fat (0.1 kg)—both measurements were measured using InBody 230 (Biospace Co., Ltd., Seoul, Republic of Korea).

Physical performance was tested using: counter movement jump (CMJ) (0.1 cm); squat jump (SJ) (0.1 cm); counter movement jump left leg (CMJLL) (0.1 cm); counter movement jump right leg (CMJRL) (0.1 cm)—these four motor tests were assessed using the Probotics tensio platform 8602 Esslinger Court Huntsville Al 25802. Vertical jump – (VJ) (0.1 cm)—was estimated using a Vertec vertical jump device TM.

Aerobic endurance was tested with 20 m shuttle run (mL/kg/min), and indirectly obtained VO_2_ max value prescribed by Léger et al. [26].

### 2.2. Participants

The research was conducted on a total sample of 46 basketball players of young age from the basketball club “Student 014” from Valjevo, Serbia (average values height = 178.26 ± 7.40 cm; weight = 64.38 ± 8.37 kg and body mass index = 20.76 ± 1.48 kg/m^2^) who at the time of measurement were on average 13.88 ± 0.53 years old. The first measurement was before quarantine and the COVID-19 virus pandemic in January 2020. The second was after the quarantine period, one year later, in March 2021, when COVID measures mitigation occurred. Measurement of morphological characteristics, and physical performance was done after the obtained permit from the Sports Association of Valjevo for the anthropological research in the basketball club “Student 014” from Valjevo registered under number 110–67/20. Basketball club “Student 014” at the time of measurement was competing in the Development league of Western Serbia.

### 2.3. Testing Procedures

Counter movement jump—CMJ was performed so that all phases of the jump were connected. Participants put their hands on their hips (for the maximum possible isolation during the jump). The subject stood in an upright position for a few seconds from which they descended to the semi-squat position (legs were flexed at the knees at a 90° angle) and without stopping at the point of change of direction, they performed the maximum vertical jump. This was followed by a soft landing with a slight flexion in the knees, and regaining the starting position. The test evaluated the eccentric–concentric component of the explosiveness of the jump. The jump height was expressed in values of 0.1 cm. Squat jump—SJ was performed from a static position. The subject also had to put their hands on their hips and take an upright standing position for a few seconds from which they descended to the semi-squat position, where they rested for two seconds. The resting phase was followed by a maximum vertical jump and a landing with slight flexion in the knees. After that, they took the starting position again. The test evaluated the concentric component of the jump explosiveness, and the jump height was expressed in values of 0.1 cm. Counter movement jump left and right legs were performed from a static position on one leg. The examinee also had to put his hands on his hips. The leg with which the jump was performed was flexed at the knee at an angle of 90°, and the insulated leg was placed in a position so that the lower leg wa raised off the ground. The subject stood in an upright position for a few seconds, from which they descended to the half-squat position and without stopping at the point of changing the direction of movement, performed the maximum vertical jump on one leg. The landing was on the same leg with a slight flexion in the knee. This was followed by regaining the initial foot position. The test evaluated the concentric component of the explosiveness of the jump of one leg, and the result of the jump was expressed in values of 0.1 cm. Vertical jump—VJ was performed using a Vertec TM vertical jump measuring device (Vertec TM, Jump USA). The examinee was in front of the Vertec, and with arms fully raised above the head, they touched the highest possible point on the Vertec device where the height could be adjusted. They further lowered to the semi-squat position and made a vertical jump with a strong swing of their hands. At the highest point of the jump, the examinee moved the bar, which showed the height of the jump. The test assessed the explosive power of the lower limbs and the maximum value of the vertical jump, and the result of the jump height was expressed in values of 0.1 cm.

### 2.4. Statistical Analysis

The statistical program SPSS Statistics for windows, version 23 (Statistical package for the social sciences, SPSS Inc, Chicago, IL, USA) was used for statistical data analysis. The distribution normality test was performed using the Shapiro–Wilk test for small samples. For all obtained data, the basic parameters of descriptive statistics were calculated. Multivariate analysis of variance (Manova) was used to determine differences in the entire sample, and univariate analysis of variance was used to determine individual differences (Anova). Based on the partial eta coefficient η^2^, the strength of the manifested change was determined for each variable (effect size).

## 3. Results

Based on the average values of the tested variables in Table 1, it can be concluded that the respondents measured after the quarantine had slightly more pronounced average values of body weight and body mass index that did not differ significantly from the values measured before the quarantine, and body height values that differed significantly after quarantine. It was also observed that they achieved, on average, lower values of total muscle mass and higher values in the variable total fat. The values of the Shapiro–Wilk coefficient indicated that there was no significant deviation of the distribution of variables from the normal distribution of results in the subjects of both groups in morphological variables. Normal nutrition could be observed in both groups.

Based on the value of the multivariate Wilks’ F test and its significance, also shown in Table 1, it is concluded that there is a significant difference (*p* < 0.04) between the two measurements in basketball players of pioneer age measured before the state of emergency and after the quarantine in terms of their morphological characteristics observing the entire system of applied variables. Individual analysis of each morphological variable concluded that these differences were expressed in the variable Total muscle mass (*p* < 0.01) in favor of higher average values for the subjects before quarantine, in the variable of the total amount of fat (*p* < 0.03), and in the variable body height (*p* < 0.02) in favor of subjects measured after quarantine. Based on the partial eta squared coefficient, which indicates the level of difference, the most dominant difference was manifested in two variables that evaluate the muscle and fat content (η^2^ = 16%; η^2^ = 10%) and the variable that evaluates the longitudinal dimensionality of the skeleton (η^2^ = 12%). All three variables describe a relatively small strength of the change effect. No significant differences were observed in the remaining four analyzed variables.

In Table 2, based on the value of the multivariate Wilks’ F test and its significance, a significant difference (*p* < 0.02) can be found between the players measured before and after the quarantine in terms of their explosive strength and aerobic endurance tests. An individual analysis of each tested variable concluded that this difference was expressed in two of the six variables, namely: the counter movement jump—CMJ (*p* <0.05) and the variables 20 m Shuttle run (*p* < 0.05) in favor of poorer average values for the players assessed after the quarantine. In this case, too, based on the partial eta squared coefficient, it can be seen that the most dominant difference was also manifested in these two variables with an impact strength of some 9% (η^2^ = 9%; η^2^ = 9%) which also represented a small magnitude of the change effect. In other tested variables, worse average results were observed in the subjects tested after quarantine, but they were not significant.

## 4. Discussion

This study analyzed the condition of a sample of basketball players of young age aimed at detecting changes after the quarantine in terms of their physical performance and morphological characteristics. Based on the research results, it can be concluded that the group of basketball players differ significantly in the final compared to the initial measurement in terms of morphological characteristics. A significant difference is also present in body height, which is a direct consequence of growth and development and cannot be attributed to the effects of COVID-19. Differences are manifested in body composition in the percentage of total muscle mass and total amount of fat in the body, with poorer results in subjects who were measured after the quarantine. It should be noted that it is very important when trying to define and explain body composition and any differences in athletes/children at this age, in order to take into account the distribution of body constitution, changes in somatotype [27], and stability of individual body constitution during growth and development [28]. The changes that are present at puberty can mask the true picture of fat in relation to muscle tissue. Therefore, it would be important to consider other anthropometric measurements so that the findings of the obtained research results would be more complete. It is a very useful method of assessing morphological characteristics; namely, there are comparisons of some of these methods, so there are also differences in opinions about their use between the authors. An earlier conviction indicates that the bioelectrical impedance (BIA) method is more useful than others because it provides results at the whole-body level [29], but the assessment in an older sample of subjects must be taken into account. Contrary to this fact, Eisenmann et al. [30] indicated that the BIA method has limited application in children up to the age of 8. However, it can be said that standard anthropometric methods for defining morphological types are still more present in relation to the BIA method [31], which could not be assumed considering the ease of estimating the parameters of body composition. That fact probably indicates the weaknesses of the BIA method compared to others, although there are no significant works that indicate the validity of the obtained parameters from the BIA method.

Average values of body height are similar to the findings of Guimarães et al. [32] (177.6 ± 6.2 cm), so there are no significant changes when it comes to growth and development. It can be stated that at this age, growth happens evenly and that subjects measured after the COVID-19 pandemic are slightly higher but with significantly higher average values of total body fat, which indicates instability in adipose tissue. These research results are consistent with the findings of Gryko et al. [33]. This can be explained by the fact that adipose tissue, which normally acts as ballast when it comes to mobility, cannot always be considered useless. Namely, adipose tissue in the pre-pubertal period indicates a faster entry into the growth phase, and this is explained by the fact that cells are filled with fat, which in this case serves as fuel to make bones grow faster and more efficiently [34]. In our case, the higher subjects had higher amounts of body fat, which in this period cannot be attributed to the intensive phase of bone growth in length, but probably to the lack of movement. If we add the average values of body weight before and after the pandemic (63.44 ± 9.72 kg and 65.60 ± 6.48 kg), it can be noticed that the volume and body weight of the tested subjects after the pandemic increased slightly. In comparison with the findings of the research by Guimarães et al. [32] (66.1 ± 8.2 kg), it can be seen that there is no major deviation in body weight at this age and that our results are in line with the above-mentioned findings. The obtained data can be explained from the aspect of reduced physical activity and restriction of movement, and interruption of the training process for a period of three months because the sample was in quarantine conditions and did not have adequate training conditions due to the closure of sports facilities. With the reduction of physical activity to which they are not accustomed, their energy intake of calories certainly increased, which was reflected in the increase in the total amount of fat, and definitely in the reduction of the percentage of total muscle mass in the body. Namely, it is clear that due to the instability of the total amount of fat in the body, and slightly more pronounced average values of body weight, there was a slight change in body mass index—(BMI) (20.50 ± 1.69 (kg/m^2^) and 21.06 ± 1.15 (kg/m^2^)) in favor of higher average values of participants measured after the pandemic. This study found evidence of differences that typically characterize subjects of the same age group in terms of anthropometric scores, body composition, and physical performance measurements from previous research in youth ball sports teams [35,36,37,38]. Research by Coelho e Silva et al. [39] indicates that at this age, body weight is negatively associated with two functional indicators (jumping, multistage shuttle run) and two basketball skills (dribbling, defensive movements).

The increase in total body fat, body weight, and BMI, and decreased muscle mass was reflected in the results of the motor test used to assess the explosive power of the lower limbs counter movement jump—CMJ also in favor of lower average values measured after the quarantine. Not enough research has been done to compare the findings with certainty. The average values of CMJ in the group of participants in our study were quite similar to the results of the study by Areda et al. [40], which was conducted before the quarantine in Spain. The authors state that the average values of CMJ were 30.31 ± 3.48 cm compared to the results in our study in basketball players before the quarantine (31.12 ± 3.03 cm) and examinees tested after the quarantine (28.97 ± 4.19 cm). Based on the above, a slight decline in values can be observed compared to the subjects before the quarantine, which can be supported by the fact that there were significant changes to reduced muscle strength due to reduced activity, limited movement, and inability to conduct the training process. It should be noted that recent findings by Ramos et al. [41] indicate that vertical jump with arm swing is one of the strongest predictors of success in basketball in a sample of children under 14 in Portugal. Similar findings are made by Cui et al. [42] on a large sample of participants. The reasons for the decline in explosive power lie in the anatomical and physiological nature because these are the factors that determine motor skills. The following analogy should be followed. Namely, if anatomical–physiological factors are crucial, then there must be differences in the morphology and structure of the tissue because differences in motor skills can occur only by changing the factors that affect it. When comparing the values in the squat jump test—SJ before and after the quarantine, there is also a decrease in average values (before—29.12 ± 3.19 cm, after quarantine—28.03 ± 4.49 cm), but compared with the results of the study [40] where average values of 27.24 ± 2.91 cm were recorded, we can still talk about better average values, even after the drop in performance. Namely, after the quarantine, a drop in the results in this variable is observed, although no significant differences were found between the initial and final measurements. In this case, too, changes in the parameters of body fat, body mass, BMI, and a decrease in muscle mass in combination with a decrease in the training process lead to poorer results in the SJ test. A study conducted by Li and Aruin [43], on subjects who were added loads to determine whether there were changes in the fulcrum when doing a jump and how much it increases the activity of postural muscles, showed that there were changes in the center of gravity, and electromyography showed an increased contraction of the trunk muscles and lower limbs. The contractions were more pronounced when the load increased. The results finally showed that changes in body weight destabilize the body, which is why it is necessary for the CNS (central nervous system) to compensate it by additional engagement of postural muscles in order to stabilize the body during an explosive jump. Furthermore, recent research by Gryko [44] indicates a negative link between body height and body weight with vertical jump tests in the same sample of Polish basketball players. The opposite findings are from previous research by Delextrat and Cohen [45] and indicate that the results of men’s vertical jump performance are unrelated to body fat content. That body fat, volume, and body weight are very important in testing physical performance, such as jumping, and that some tests are inconsistently applied is indicated by some research [46,47]. Namely, the authors believe that it is necessary to take into account the role of the allometric relationship between the tested performance and the body size index when testing external force in rapid movement tests (jumps or sprints). In that case, the standardization of tests should be done by applying an allometric ratio that would be calculated by the formula Pn = P/Sb, where P—tested performance and Sb—body size index.

Furthermore, based on the obtained research results, it can be concluded that significant differences between the two measurements were also found during the test that assessed aerobic endurance in favor of poorer average values of subjects measured after the quarantine. These results are consistent with other findings [48,49]. Most authors also believe that as a result of the inability to perform the training process continuously, and a number of exercises that were adapted to special conditions instead of fitness training, there was a decrease in the level of aerobic endurance in young basketball players. This was directly manifested by reduced running for a long time, the inability to do endurance exercises, and the simulation of basketball games. As a result, rapid fatigue occurred during testing because the heart rate increased very quickly and reached higher values, which is a direct consequence of the negative association VO_2_max [50]. This can be explained by the fact that due to rapid fatigue and inability to realize movement, muscle acidification and poorer transport of oxygen to muscle cells occurs, which reduces aerobic endurance. Authors like Abdelkrim et al. [51] also point to the fact that running speed is significantly correlated with endurance performance. Authors Nazaraki et al. [52] state that the maximum oxygen consumption (VO_2_ max) reaches up to 42 mL/kg/min in basketball players, which is quite similar to the results obtained before quarantine (41.60 ± 5.54 mL/kg)/min), but significantly less after quarantine (38.42 ± 4.84 mL/kg/min). Some authors believe that the best way to increase physical fitness is integrated training exercises on the basketball court [53,54,55]. The authors recommend 4 × 4 min play, two versus two, with 3 min passive recovery. Then 3 × 6 × 20 m exercise with ball, 20 s shuttle running exercises, and 4 min recovery. They also believe that the type of increase in physiological load could be a simulation of a match with a smaller number of players with an increase in time. Basketball is considered to be a very intense sport, with players changing places on the court approximately every one to three seconds, moving in different directions, which directly affects the relationship between high and low-intensity aerobic endurance, and these tactical ideas are starting to be introduced in sports clubs with children from the age of twelve [56,57,58]. Thus, in particular, high-intensity movements such as intense running, sprints and jumps make up about 8.8% of the total time of the match [59]. Therefore, a focus on aerobic endurance would increase the physical condition of basketball players, but one should also take into account geographical differences in activity requirements (distance and frequency) and physiological responses [60,61].

The decline in physical activity during the COVID-19 pandemic in 2020, caused by the introduction of restrictive measures of movement and interruption of the training process by younger age basketball players in this study, ultimately resulted in weight gain, an increase in total body fat, and a decrease in total muscle mass. This directly led to a decrease in the level of the explosive power of the lower limbs as well as a decrease in aerobic endurance. In the end, based on the analysis presented so far, it can be concluded that the hypothesis can be fully accepted. The limitations of the study can be reflected in the number of participants that was followed from the initial to the final measurement because the pandemic caused the withdrawal of a number of children from the final measurement. Therefore, the lack of the study would be reflected in the impossibility of monitoring the same number of players before the SARS-CoV-2 virus pandemic, where cleaner measurement results would be obtained, and the external validity of the study would not be threatened due to the possibility of greater generalization of pioneer-age basketball players. The authors believe that it would be useful to devise a specially programmed training process for aerobic endurance in quarantine conditions (on a treadmill) in order to avoid its decrease. In addition, trainers should design special strength training and vertical jump training. Moreover, it would be very useful to organize online meetings with all athletes at least four times a week to give them advice on the training process and useful tips on diet, calorie intake, and consumption. Nevertheless, we should not ignore recent research by authors Arslan et al. [62], in which two different training methods were compared, the first 6-week small-sided games training (SSGs) and high-intensity interval training (HIIT), which indicate that both methods provide positive physical adaptations with an additional improvement in the technical skills of young basketball players of the pioneering aged 14 years. Only with such measures taken and preparation for possible new situations, could the decline in characteristics crucial for basketball and anthropological characteristics be reduced.

## 5. Conclusions

The authors conclude that the consequences of the quarantine and pandemic of the SARS-CoV-2 virus were definitely negative, as higher values of body fat and lower values of the percentage of total muscle mass were observed. In addition, a lower level of explosive power in the lower limbs was observed, accompanied by lower aerobic endurance of basketball players.

## Figures and Tables

**Table 1:** Descriptive statistics and differences in morphological characteristics and body composition.

Variable	Before Quarantine		After Quarantine				
	AM ± S	SWp	AM ± S	SWp	f	*p*	η^2^
Body height (cm)	175.30 ± 7.64	0.81	181.22 ± 7.17	0.51	5.15	0.02	0.12
Body weight (kg)	63.44 ± 9.72	0.21	65.50 ± 6.48	0.06	0.69	0.41	0.02
Total muscle mass (%)	35.98 ± 1.88	0.97	34.37 ± 1.83	0.25	8.45	0.01	0.16
Total fat (%)	19.22 ± 5.48	0.92	22.81 ± 5.61	0.10	4.78	0.03	0.10
Body mass index (kg/m^2^)	20.50 ± 1.69	0.59	21.06 ± 1.15	0.68	1.65	0.21	0.04
F = 2.40 *p* < 0.04							

Note. AM—mean; S—standard deviation; SWp—level of statistical significance of Shapiro–Wilk coefficient; f—univariate f test; *p*—level of statistical significance of the univariate f test; η^2^—partial eta squared coefficient on test strength and sufficient sample size; F—multivariate Wilks’ F test; *p*—statistical significance of multivariate F test.

**Table 2:** Descriptive statistics and differences in motor skills and aerobic endurance.

Variable	Before Quarantine		After Quarantine				
	AM ± S	SWp	AM ± S	SWp	f	*p*	η^2^
CMJ (cm)	31.12 ± 3.03	0.14	28.97 ± 4.19	0.87	4.07	0.05	0.09
SJ (cm)	29.92 ± 3.19	0.06	28.03 ± 4.49	0.13	2.80	0.10	0.06
VJ (cm)	47.17 ± 4.21	0.13	46.40 ± 5.09	0.87	0.32	0.57	0.01
CMJ LL(cm)	21.58 ± 3.31	0.28	22.56 ± 3.50	0.54	0.45	0.51	0.01
CMJRL (cm)	22.78 ± 3.65	0.48	22.07 ± 3.19	0.33	0.48	0.49	0.01
20 m Shuttle run (mL/kg/min)	41.60 ± 5.54	0.20	38.42 ± 4.84	0.22	4.19	0.05	0.09
F = 2.98 *p* < 0.02							

Note. AM—mean; S—standard deviation; SWp—level of statistical significance of Shapiro–Wilk coefficient; f—univariate f test; *p*—level of statistical significance of the univariate f test; η^2^—partial eta squared coefficient on test strength and sufficient sample size; F—multivariate Wilks’ F test; *p*—statistical significance of the multivariate F test.

## Data Availability

Data available on reasonable request to the corresponding author (vladan.pelemis@uf.bg.ac.rs).
